# Aortic volume determines global end-diastolic volume measured by transpulmonary thermodilution

**DOI:** 10.1186/s40635-019-0284-8

**Published:** 2020-01-02

**Authors:** Aleksej Akohov, Christoph Barner, Steffen Grimmer, Roland CE Francis, Stefan Wolf

**Affiliations:** 1 Department of Anesthesiology and Intensive Care Medicine (CCM/CVK), Charité – Universitätsmedizin Berlin, Freie Universität Berlin, Humboldt-Universität zu Berlin, and Berlin Institute of Health, Berlin, Germany; 2 Department of Neurosurgery, Charité Campus Mitte, Charité – Universitätsmedizin Berlin, Freie Universität Berlin, Humboldt-Universität zu Berlin, and Berlin Institute of Health, Berlin, Germany; 3 Department of Anesthesiology, Vivantes Klinikum Neukölln, Vivantes Netzwerk für Gesundheit, Berlin, Germany

**Keywords:** Aorta, Aortic volume, Global end-diastolic volume, GEDV, GEDVI, Transpulmonary thermodilution, Vena cava, Vena cava volume

## Abstract

**Background:**

Global end-diastolic volume (GEDV) measured by transpulmonary thermodilution is regarded as indicator of cardiac preload. A bolus of cold saline injected in a central vein travels through the heart and lung, but also the aorta until detection in a femoral artery. While it is well accepted that injection in the inferior vena cava results in higher values, the impact of the aortic volume on GEDV is unknown. In this study, we hypothesized that a larger aortic volume directly translates to a numerically higher GEDV measurement.

**Methods:**

We retrospectively analyzed data from 88 critically ill patients with thermodilution monitoring and who did require a contrast-enhanced thoraco-abdominal computed tomography scan. Aortic volumes derived from imaging were compared with GEDV measurements in temporal proximity.

**Results:**

Median aortic volume was 194 ml (interquartile range 147 to 249 ml). Per milliliter increase of the aortic volume, we found a GEDV increase by 3.0 ml (95% CI 2.0 to 4.1 ml, *p* < 0.001). In case a femoral central venous line was used for saline bolus injection, GEDV raised additionally by 2.1 ml (95% CI 0.5 to 3.7 ml, *p* = 0.01) per ml volume of the vena cava inferior. Aortic volume explained 59.3% of the variance of thermodilution-derived GEDV. When aortic volume was included in multivariate regression, GEDV variance was unaffected by sex, age, body height, and weight.

**Conclusions:**

Our results suggest that the aortic volume is a substantial confounding variable for GEDV measurements performed with transpulmonary thermodilution. As the aorta is anatomically located after the heart, GEDV should not be considered to reflect cardiac preload. Guiding volume management by raw or indexed reference ranges of GEDV may be misleading.

## Introduction

Transpulmonary thermodilution is commonly used and recommended in current guidelines for the management of critically ill patients with cardiovascular instability to assess cardiac output (CO) and volume status [1, 2]. The parameter global end-diastolic volume (GEDV), a hypothetical volume assuming all cardiac chambers being simultaneously in diastole, is considered to reflect cardiac preload [3]. Michard et al. described that GEDV indexed to body surface area (GEDVI) more adequately predicted volume responsiveness in patients with septic shock compared with the central venous pressure [4]. In a prospective randomized trial, Goepfert et al. found that guidance with an algorithm including GEDVI reduced complications and length of ICU stay in patients after cardiac surgery [5]. Kaneko et al. identified GEDVI as an important contributor to elevated extravascular lung water (EVLW) in patients with ARDS [6].

However, it was recently shown that GEDVI did not reflect even markedly enlarged left-ventricular end-diastolic volumes measured by cardiac angiography [7]. Furthermore, reference values for GEDVI proposed by expert opinion vary and a reference range applicable to all subjects was repeatedly questioned [8–10]. A meta-analysis including 64 studies recognized significantly higher mean GEDVI in septic patients compared with patients undergoing major surgery and concluded the need to adapt therapeutic targets for different patient populations [8]. Huber et al. noticed a dependence of GEDV on age, sex, body height, and body weight in patients in a medical intensive care unit and proposed sex-specific formulas to alleviate the problem of indexation [9]. A prospective observational trial found a large inter-individual variability of GEDV and GEDVI and hypothesized that the aortic volume might be the source of the observed heterogeneity [10]. This potential explanation was based on the fact that the cold saline bolus injected for measurement must transit the aorta to reach the temperature detector placed in a femoral artery. It is well known that the aortic size increases with age and is sex dependent [11]. Patients with an aortic aneurysm present with higher GEDVI values [12]. However, neither the theoretical derivation nor contemporary reviews of GEDV and GEDVI measured by transpulmonary thermodilution do consider the aortic volume [13–16].

In the present study, we investigate the hypothesis of a relationship between aortic volume and GEDV.

## Methods

### Study population

**Fig. 1:**
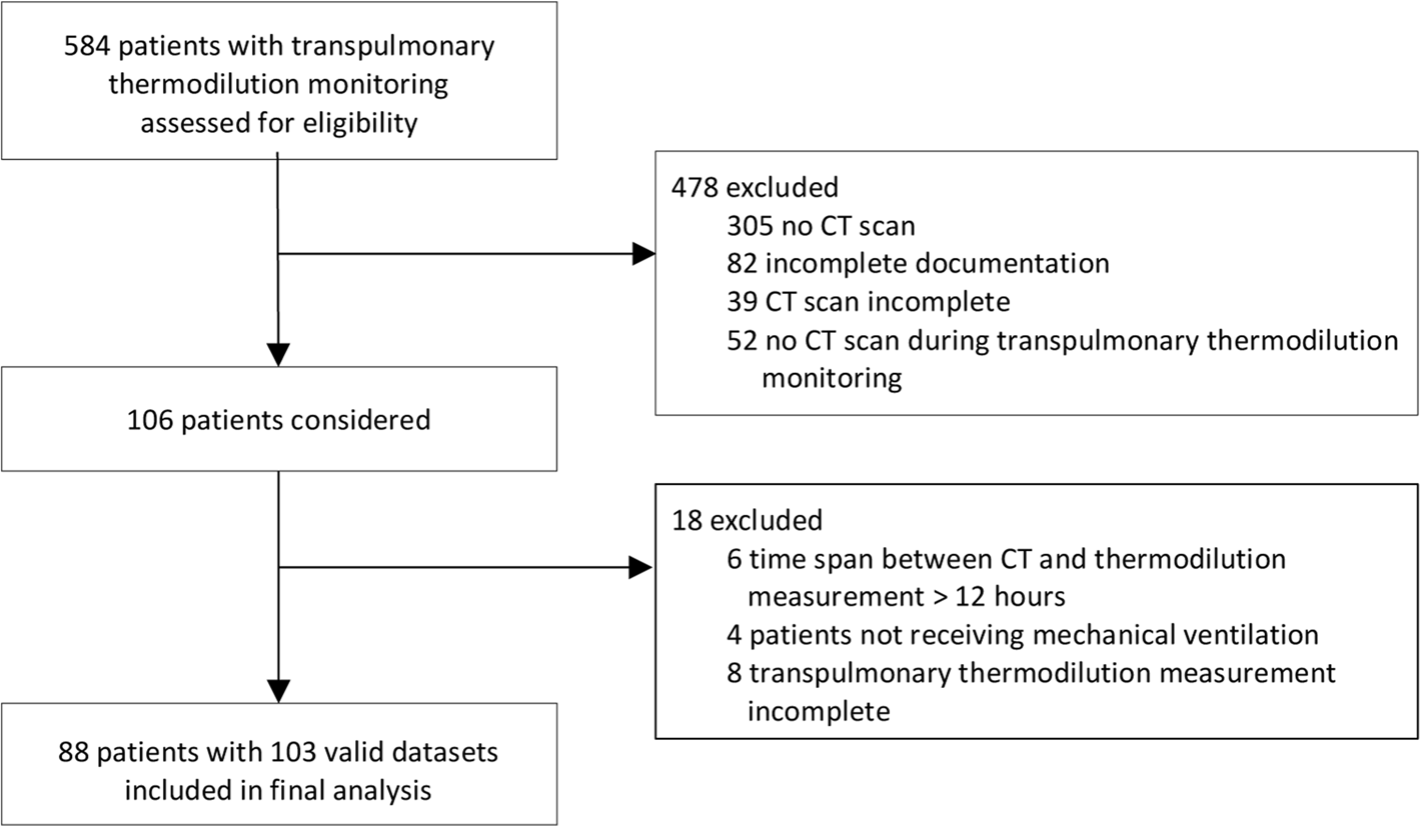
Flow diagram of patient identification. CT computed tomography

The study was approved by the Ethics Committee of Charité - Universitätsmedizin Berlin (vote EA 1/084/13). The study was performed at the Interdisciplinary Neurointensive Care Unit of Charité – Universitätsmedizin Berlin at Charité Campus Virchow, with inclusion from January 2009 to December 2016. We identified subjects who had monitoring with transpulmonary thermodilution implemented. Additionally, patients were required to have received a contrast-enhanced CT scan of the thorax and abdomen, either as screening for injury after trauma, but also in search of a septic focus. We selected patients with mechanical ventilation and an arbitrarily chosen time difference of maximum 12 h between CT scan and thermodilution measurement (Fig. 1).

#### Transpulmonary thermodilution measurements

As usual in transpulmonary thermodilution, iced saline was injected via a central venous line and the resulting thermal signal was detected by a thermodilution catheter (PVPK 2015 L20-A) in a femoral artery. Both catheters were connected to a PiCCO2 monitor (Pulsion Medical Systems, Munich, Germany).

Cardiac output (CO) is derived from the area under the curve by the Stewart-Hamilton formula [17]. The thermal signal may be characterized further by mean transit time (MTt) and downslope time (DSt), the inverse of its rate of decay [15, 18]. MTt times CO equals the distribution volume of thermal indicator, the intrathoracic thermal volume (ITTV). In a series of sequentially traversed volumes, the largest one determines the DSt [13]. In case of transpulmonary thermodilution, the largest thermal compartment is assumed to be the lung, resulting in the pulmonary thermal volume PTV = CO × DSt. The difference between ITTV and PTV equals the GEDV, which may be calculated as:

$$ \mathrm{GEDV}=\left(\mathrm{CO}\times \mathrm{MTt}\right)-\left(\mathrm{CO}\times \mathrm{DSt}\right) $$

CO, MTt, DSt, GEDV, and EVLW were obtained from the average of a series of at least three venous injections of 20 ml of iced saline [19], with outliers (± 3 SD) discarded. All thermodilution data were extracted from archived log files of the PiCCO2 devices. As suggested by the manufacturer, GEDVI was calculated by dividing GEDV by body surface area based on predicted body weight.

Of note, in transpulmonary thermodilution, the volume between the aortic valve and the detector in a femoral artery is obviously traversed by the cold indicator bolus. Therefore, measured GEDV may be split in a venous volume, a central part—the volume of interest as surrogate for cardiac preload—and the aortic volume:

$$ {\mathrm{GEDV}}_{\mathrm{measured}}={\mathrm{GEDV}}_{\mathrm{venous}}+{\mathrm{GEDV}}_{\mathrm{central}}+{\mathrm{GEDV}}_{\mathrm{aortic}} $$

The venous part may be assumed to be zero in case of a central venous line in the superior vena cava. However, the aortic part of GEDV remains inevitably included in transpulmonary thermodilution measurements.

#### Image analysis

**Fig. 2:**
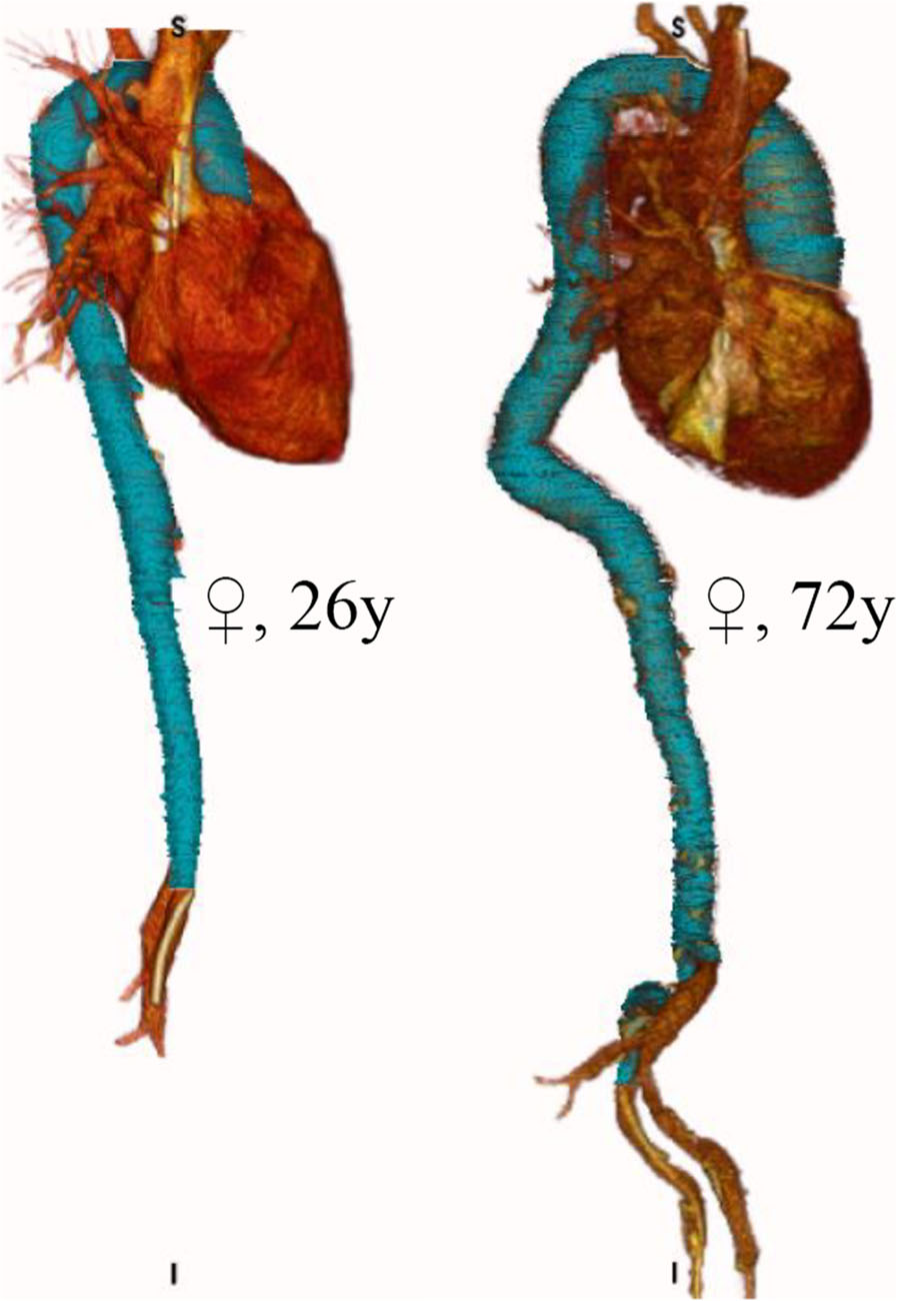
Representative reconstructed three-dimensional sagittal computed tomography images of the heart and the aorta. Left side is from a 26-year-old female with meningoencephalitis and septic shock, 59 kg, 170 cm. GEDV 502 ml, GEDVI 293 ml/m^2^. Right side shows data from a 72-year-old female with aneurysmal subarachnoid hemorrhage, 165 cm, 78 kg. GEDV 1263 ml, GEDVI 787 ml/m^2^. 3D rotational images are provided in the electronic supplements (see Additional files 1 and 2). Aortic volume, defined as the volume of the aorta between the left coronary artery and the tip of the femoral catheter, is visualized in blue. Proportions reflect real dimensions. Note the difference in size and shape of the aortic volume

Contrast-enhanced thoracic-abdominal CT scans were retrieved from the Picture Archive and Communication System GEPACS (Centricity PACS 3.2 RA 1000 Workstation, GE Healthcare, Chicago, USA). Post-processing of the images was performed with Osirix® MD 6.5.2 (Pixmeo SARL, Geneva, Switzerland). The aorta was identified on axial slides and marked manually as region of interest (ROI) [20, 21]. The resulting sequence of interconnected ROIs together with the slice width was used for volume calculation, with the left coronary artery and the tip of the transpulmonary thermodilution catheter in the femoral artery as longitudinal boundaries. This reconstructed volume is referred to as “aortic volume” (Fig. 2, Additional files 1 and 2). For length determination, a central path was marked manually. Diameters were calculated from cross-sectional areas assuming circular boundaries. When a femoral central venous catheter was present, reconstruction of the volume of the inferior vena cava was performed likewise, using the tip of the catheter and the right atrium as boundaries. In patients with a subclavian or jugular central venous catheter, the correct position of its tip is at the entrance of the right atrium. Consequentially, the additional volume of the vena cava relevant for thermodilution measurements was assumed to be zero.

#### Statistical analysis

Statistical computation was performed with R 3.4.3 (R Core Team, R Software Foundation, Vienna, Austria, 2018). Results are given as median and interquartile range (IQR) or with mean and corresponding 95% confidence intervals (95% CI), as appropriate. No imputation was performed for missing data. Regression analysis was performed with robust linear regression (R package *robustbase*, version 0.93-5) to account for heteroscedasticity and skewness. Biometric parameters (age, sex, body height, and weight) were investigated simultaneously to account for partial correlation using multivariate models. Mixed effect models to correct for repeated CT measurements in few patients proved not to be superior by the minimized Akaike Information Criterion (AIC) [22]. Therefore, in favor of parsimony, all measurements were regarded as independent. Explained variance is given by adjusted *R*^2^. *p* values less than 0.05 were considered significant.

## Results

### Data description

We identified 103 CT scans in 88 patients meeting the inclusion criteria (Fig. 1). Demographic data of the patients are shown in Table 1. ICU scores, vasoactive drugs, ventilation parameters, and location of central venous catheters are shown in Table 2. Included in this table are time differences and fluid balance between CT scanning and thermodilution measurements. Data of CT scans and transpulmonary thermodilution measurements are given in Table 3.

**Table 1:** Patient characteristics

Patients	All	Female	Male
*n* (%)	88 (100)	32 (36.4)	56 (63.6)
Age, year	57 (42–68)	52 (40–70)	59 (45–67)
Weight, kg	80 (70–90)	65 (60–80)	85 (75–94)
Body height, m	1.74 (1.68–1.80)	1.65 (1.60–1.70)	1.8 (1.73–1.83)
BMI, kg/m^2^	26 (23–29)	25 (22–29)	26 (24–29)
Reason for ICU admission, *n* (%)			
Traumatic brain injury	22 (25.0)	2 (6.3)	20 (35.7)
Spontaneous intracranial hemorrhage	16 (18.2)	1 (3.1)	15 (26.8)
Aneurysmal subarachnoid hemorrhage	14 (15.9)	9 (28.1)	5 (8.9)
Infections of the central nervous system	8 (9.1)	5 (15.6)	3 (5.4)
Sepsis	6 (6.8)	4 (12.5)	2 (3.6)
Infarct	6 (6.8)	2 (6.3)	4 (7.1)
Tumor	4 (4.5)	4 (12.5)	0 (0)
Respiratory failure	3 (3.4)	0 (0)	3 (5.4)
Other	9 (10.2)	5 (15.6)	4 (7.1)
In-hospital mortality, *n* (%)	39 (44.3)	16 (50)	23 (41.1)

*BMI* body mass index, *ICU* intensive care unit. Data are presented as median (interquartile range) or number (frequency in percent)

**Table 2:** Clinical data at time of CT and thermodilution measurement, respectively

	All	Female patients	Male patients
*n* (%)	103 (100)	38 (36.9)	65 (63.1)
ICU scoring			
APACHE II	26 (21–31)	27 (20–32)	25 (22–30)
SAPS II	56 (45–67)	54 (43–63)	58 (46–68)
SOFA	11 (9–13)	11 (9–14)	11 (9–13)
Patients receiving catecholamines, *n* (%)			
Adrenaline	4 (3.9)	1 (2.6)	3 (4.6)
Dobutamine	7 (6.8)	2 (5.3)	5 (7.7)
Norepinephrine	101 (98.1)	37 (97.4)	64 (98.5)
Norepinephrine dose at time of TD measurement, μg/kg/min	0.27 (0.11–0.52)	0.32 (0.12–0.53)	0.20 (0.11–0.47)
Norepinephrine dose at time of CT, μg/kg/min	0.26 (0.11–0.50)	0.30 (0.11–0.51)	0.25 (0.11–0.50)
Patients receiving another cardiovascular agent, *n* (%)			
Enoximone	10 (9.7)	3 (7.9)	7 (10.8)
Nitroglycerine	3 (2.9)	2 (5.3)	1 (1.5)
Vasopressin	2 (1.9)	1 (2.6)	1 (1.5)
Patients receiving more than one cardiovascular agent, n (%)	21 (20.4)	7 (18.4)	14 (21.5)
Parameters of mechanical ventilation at time of thermodilution measurement			
PEEP, mmHg	10 (9–13)	10 (9–12)	10 (9–13)
f, 1/min	21 (18–25)	20 (17–24)	22 (18–26)
VT, l	0.49 (0.40–0.55)	0.41 (0.33–0.49)	0.51 (0.46–0.57)
Parameters of mechanical ventilation at time of CT			
PEEP, mmHg	10 (9–13)	10 (9–13)	11 (9–13)
f, 1/min	21 (17–25)	21 (17–25)	22 (18–25)
VT, l	0.49 (0.40–0.55)	0.40 (0.36–0.49)	0.52 (0.46–0.56)
Time span between CT and thermodilution measurement, hours	1 (-1–3)	0 (-1–3)	1 (-1–3)
Fluid balance between CT and thermodilution measurement, ml	-10 (-198–258)	0 (-94–434)	-31 (-289–128)
Continuous veno-venous hemodialysis, *n* (%)	20 (19.4)	6 (15.8)	14 (21.5)
CVC position			
V. jugularis, *n* (%)	48 (46.6)	20 (52.6)	28 (43.1)
V. subclavia, *n* (%)	40 (38.8)	13 (34.2)	27 (41.5)
V. femoralis, *n* (%)	15 (14.6)	5 (13.2)	10 (15.4)
Aortic aneurysm, *n* (%)	2 (1.9)	0 (0)	2 (3.1)
Status post-OAR or EVAR, *n* (%)	5 (4.9)	0 (0)	5 (7.7)

*APACHE II* acute physiology and chronic health evaluation II, *CVC* central venous catheter, *EVAR* endovascular aortic aneurysm repair, *ICU* intensive care unit, *OAR* open aortic repair, *SAPS II* simplified acute physiology score, *SOFA* sequential organ failure assessment. Data are presented as median (interquartile range) or number (frequency in percent)

**Table 3:** Aortic and vena cava length and volume derived from CT scans and physiologic values from transpulmonary thermodilution measurements

	All	Female patients	Male patients
*n* (%)	103 (100)	38 (36.9)	65 (63.1)
Aortic length, cm	55.2 (51.0–60.2)	50.8 (47.5–56.6)	56.4 (53.8–61.5)
Aortic volume, ml	194 (147–249)	158 (126–207)	213 (169–287)
Vena cava length (femoral CVC), cm	32.1 (27.8–33.4)	32.5 (31.1–38.1)	28.6 (21.5–32.6)
Vena cava volume (femoral CVC), ml	127 (93–155)	162 (150–188)	98 (77–122)
HR, 1/min	86 (74–103)	84 (74–100)	89 (71–105)
MAP, mmHg	83 (73–93)	84 (74–96)	83 (72–92)
CO, l/min	6.4 (5.3–7.8)	5.6 (4.9–6.8)	6.8 (5.8–8.2)
CI, l/min/m^2^	3.3 (2.8–4.0)	3.1 (2.6–3.9)	3.4 (2.9–4.2)
GEDV, ml	1306 (1104–1569)	1129 (990–1283)	1437 (1169–1658)
GEDVI, ml/m^2^	730 (627–871)	709 (627–840)	738 (628–894)
EVLW, ml	603 (510–781)	540 (462–619)	649 (528–829)
EVLWI, ml/kg	9.7 (7.8–11.6)	9.9 (8.7–11.5)	9.3 (7.7–11.7)
MTt, s	20.9 (17.7–27.3)	19.4 (17.4–27.3)	21.1 (17.9–27.4)
DSt, s	8.4 (7.2–11.3)	8.4 (7.2–10.9)	8.5 (7.2–11.3)

*CI* cardiac index, *CO* cardiac output, *CVC* central venous catheter, *EVLW* extravascular lung water, *EVLWI* extravascular lung water index, *GEDV* global end-diastolic volume, *GEDVI* global end-diastolic volume index, *HR* heart rate, *MAP* mean arterial pressure, *DSt* down slope time, *MTt* mean transit time. All data is presented as median (interquartile range)

### Aortic volume

Median aortic volume, measured from the aortic valve to the tip of the femoral artery catheter, was 158 ml (IQR 126 to 207 ml) in females and 213 ml (IQR 169 to 287 ml) in males (*p* < 0.001). Aortic volume increased by 2.3 ml (95% CI 1.7 to 2.8 ml, *p* < 0.001) per year of patient age. Aortic volume showed no significant relationship to body height (*p* > 0.05), but increased by 1.2 ml (95% CI 0.3 to 2.2 ml, *p* = 0.009) per kg of patient body weight. Measurements of aortic volume had a coefficient of repeatability of 2.1%.

### Measurements of inferior vena cava

Fifteen measurements were performed with a femoral central venous line. In two patients, we were unable to unequivocally identify the upper boundary of the vena cava at the level of the diaphragm due to enlarged hepatic veins. Thus, an accurate and reproducible volume calculation was impossible. In the remaining 13 patients, median volume of the inferior vena cava was 127 ml (IQR 93 to 155 ml). Analysis of relationships with age, sex, height, and weight was not considered meaningful due to the low number of patients.

### Dependencies of GEDV and GEDVI on biometric parameters

Median GEDV in all patients was 1306 ml (IQR 1104 to 1569 ml). Median GEDVI was 730 ml/m^2^ (IQR 627 to 871 ml/m^2^).

GEDV increased by 7.4 ml (95% CI 4.1 to 10.7 ml, *p* < 0.001) per year of patient age. Per kilogram increase in body weight, GEDV increased by 5.2 ml (95% CI 1.7 to 8.6 ml, *p* = 0.003). After correction for age and weight, GEDV showed no significant dependency on height and sex. These relationships persisted after indexing GEDV by body surface area based on predicted body weight. GEDVI increased by 4.2 ml/m^2^ (95% CI 2.3 to 6.2 ml/m^2^, *p* < 0.001) per year of age and by 2.8 ml/m^2^ (95% CI 0.9 to 4.7 ml/m^2^, *p* = 0.004) per kg body weight, while height and sex showed no significant relationship.

In patients with a femoral central venous line, GEDV was 438 ml (95% CI 235 to 641 ml, *p* < 0.001) larger than in patients with jugular or subclavian central venous catheter. Likewise, GEDVI was 230 ml/m^2^ (95% CI 89 to 370 ml/m^2^, *p* = 0.002) larger in patients with a femoral venous line.

Time differences, fluid balances, changes in ventilator settings, or the level of vasoactive drugs between thermodilution measurements and CT scans were without significant impact on GEDV (*p* > 0.05 for each comparison). GEDV measurements showed a coefficient of repeatability of 4.3%.

### Dependence of GEDV on central venous and aortic volume

A total of 38.4% of the variance of GEDV was explained by patient-specific biometric characteristics including age, sex, body weight, and body height. We then sequentially added the volumes of either the vena cava, the aorta, or both to this initial model. Inclusion of the volume of the vena cava raised the explained variance of GEDV to 47.8%. After adding the aortic volume to the basic model instead of the volume of the vena cava, explained GEDV variance was 59.3%. Combining both aortic and venous volume led to an explained variance of GEDV of 63.8%. In each model where the aortic volume was included, all biometric parameters lost their significance (Table 4).

**Table 4:** Statistical significance of confounding variables for GEDV

Statistical model	(1) Basic model: biometric descriptors	(2) Basic model + V. cava	(3) Basic model + aorta	(4) Basic model + aorta + V. cava
Age	*p < 0.001*	*p < 0.001*	*p* > 0.05	*p* > 0.05
Male sex	*p* > 0.05	*p* > 0.05	*p* > 0.05	*p* > 0.05
Height	*p* > 0.05	*p* > 0.05	*p* > 0.05	*p* > 0.05
Weight	*p = 0.003*	*p = 0.002*	*p* > 0.05	*p* > 0.05
Aortic volume			*p < 0.001*	*p* < *0.001*
Vena cava volume		*p = 0.003*		*p* = *0.009*
Explained variance	38%	48%	59%	64%
Difference to previous model		*p* *<* *0.001*	*p* < *0.001*	*p* < *0.001*

Multivariate statistical models investigate (1) the dependence of GEDV values on age, male sex, height, and body weight (basic model); (2) the biometric parameters from the basic model and additionally the volume of the vena cava; (3) the parameters from the basic model and additionally the aortic volume; and (4) the parameters from the basic model with biometric descriptors and both vena cava volume and aortic volume. Values in italics indicate significance

### Analysis of GEDV components

**Fig. 3:**
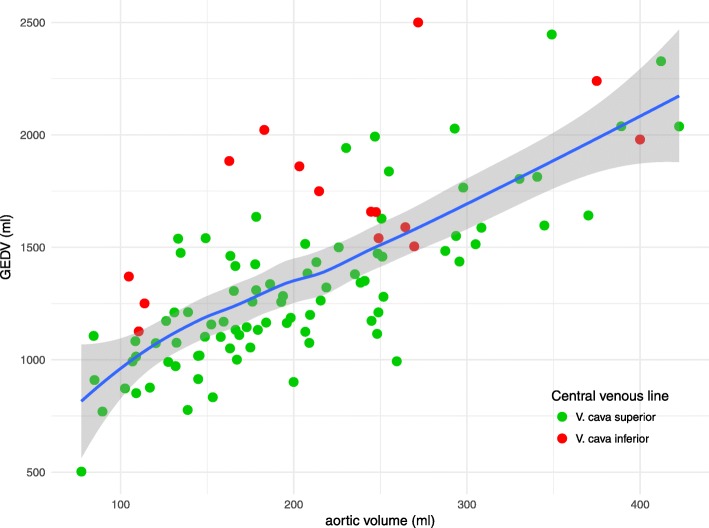
Relationship of global end-diastolic volume (GEDV) and aortic volume. Blue line indicates the regression line, with its 95% confidence interval marked in grey. Green dots represent measurements with central venous lines placed in the vena cava superior, red dots in the vena cava inferior

In the final regression model including both aortic volume and the volume of the vena cava, GEDV increased by 3.0 ml (95% CI 2.0 to 4.1 ml, *p* < 0.001) per ml of aortic volume and by 2.1 ml (95% CI 0.5 to 3.7 ml, *p* = 0.01) per ml of vena cava volume. Plotting the data suggested a linear relationship between the aortic volume and GEDV (Fig. 3).

Measured GEDV consists of a “venous,” a “central,” and an “aortic” part (see Methods). Assuming the linear relationships found above allowed for estimation of single proportions of GEDV. The aortic part was in median 49% (IQR 40 to 58%) of measured GEDV. In case of a femoral central venous line, the venous part estimated in median to 14% (IQR 14 to 17%). The central part was in median 50% (IQR 40 to 57%) of measured GEDV. The central and venous parts did not depend on biometric parameters, while the aortic part had significant relationships with age and weight (*p* < 0.001 and *p* = 0.009, respectively).

To get further insight, we examined the influence of aortic volume on the different variables required for GEDV calculation: MTt, DSt, and CO. The largest impact was on MTt, with 3.5 s (95% CI 1.4 to 5.5 s, *p* = 0.001) per 100 ml of aortic volume. Additionally, MTt showed a hyperbolic decline with raising values of CO (*p* < 0.001). CO was larger in patients with higher aortic volume, 0.5 l/min (95% CI 0 to 1 l/min, *p* = 0.041) per 100 ml aortic volume. DSt enlarged by 0.8 s (95% CI 0 to 1.7 s, *p* = 0.048) per 100 ml aortic volume.

## Discussion

As main finding, we confirmed the hypothesized relationship between GEDV and aortic volume. Aortic volume determines the value of GEDV to a larger extent than any biometric parameter, including a patient’s age, sex, body weight, and height.

### Relevance of central venous and aortic volumes for GEDV measurement

It is well accepted and confirmed by our data that femoral central venous lines should be accounted for when interpreting GEDV measurements [23–25]. However, our results show that the aortic volume had an even larger, predominant influence by explaining roughly 60% of GEDV variance. As the aortic volume is anatomically placed *after* the heart, our findings challenge the view of GEDV as a cardiac *preload* parameter.

It is important to mention that our measurements of the aortic diameter, length, and volume as well as estimated aortic mean transit times are in line with published data [11, 26–30].

### Analysis of influences on thermal volume

Contrast bolus traverse through the aorta led to a larger increase in GEDV than expected by considering plain aortic volume. Two interacting causes may be suggested. First, theory of single-indicator transpulmonary thermodilution requires a closed circulation between injection and detection site [14]. Thoracic and abdominal branches of the aorta invalidate this prerequisite. Second, the flow along the aorta is not laminar but turbulent and helical [31–33]. Both potential causes would challenge the assumption of CO times MTt being equal to the traversed volume.

In patients with a femoral central venous line, similar considerations concerning the necessary prerequisites apply. The vena cava inferior has influx from abdominal and hepatic veins, thus not resembling a closed system as required for calculation of GEDV from the thermodilution curve.

### Concerns against indexing GEDV

Indexing of a physiological parameter intends to remove inter-individual variations to facilitate comparison between patients and derive normal ranges. From a mathematical point of view, indexing represents a linear regression, which may be defined by two points only. One is the mean of the parameter to be indexed and the mean of the index. The second point is the origin, where both the parameter and the index are zero, usually far away from physiologic ranges. Therefore, the slope of the regression line is mainly determined by the origin as a gross outlier. This may lead to the removal of existing correlations, but also generation of correlations not present in the original data [34–36].

Obviously, indexing GEDV can be performed numerically, but this does not imply that the result is meaningful. The relevant confounder of GEDV, the aortic volume, is cumbersome to achieve and usually not known. Therefore, indexing by aortic volume is not applicable. In current practice, GEDV indexation is performed with predicted body surface area derived from height. In our data, height was no significant confounder. In contrast, a dependency of GEDV on age and weight was present before, but also after indexing by predicted body surface area. Furthermore, the central part of GEDV had no relationship with any biometric parameter, while the aortic part was dependent on age and weight. The ratio between both parts varies from patient to patient. The quest for reference ranges of GEDV is further complicated when femoral central venous lines are taken into account. Therefore, there is little to support a scientifically validated and clinically useful indexation.

### Clinical implications

We interpret our data that the numeric value of GEDV reflects the intravascular volume status of a patient, with preload being a minor contributor and not the dominant part. Future, prospective work may address the impact of volume loading, vasopressors, or mechanical ventilation on venous, central, and aortic components of GEDV. It is likely that this impact is different on each component, given that a controlled volume loss affects the diameter of the vena cava more than that of the abdominal aorta [37]. However, current transpulmonary thermodilution technology does not allow to distinguish between the different parts of measured GEDV. Its value is partially dependent on the aortic volume, which is itself being associated with age, sex, and weight. As a consequence, any treatment decision aiming for standardized normal values of GEDV/GEDVI may be beneficial in one patient but detrimental in another. Variations between successive measurements may have clinical importance [4], but require further study, in our opinion.

### Limitations

Missing knowledge of the effect size rendered planning of a prospective study impossible. However, electronic recording guaranteed data accuracy and we are unaware of any systematic bias concerning patient selection.

Our population was treated in a neurosurgical ICU. While this may be considered a limitation, we want to point out that subjects presented with hemodynamic instability due to various causes, were on vasopressors and required mechanical ventilation. In our opinion, this reflects a typical scenario where transpulmonary thermodilution monitoring may be applied.

Central venous lines used were multi-lumen catheters of different brands. Per clinical standard, we mount the venous thermistor for transpulmonary thermodilution on the side arm of the first 3-way stopcock on the distal lumen. Occasional use of a different ports or failure of correct placement of the catheter tip at the entrance of the right atrium in case of jugular or subclavian central venous lines may have induced a minor error we were unable to correct for.

## Conclusion

We provide evidence that the aortic volume mainly accounts for the variability of GEDV measured by single-indicator transpulmonary thermodilution with a femoral arterial line. Therefore, GEDV should not be considered to reflect the cardiac preload status of a patient. Furthermore, we were unable to provide a scientific physiological rationale for indexing GEDV. As a consequence, guiding individual volume therapy by reference ranges of GEDV or GEDVI may be misleading.

## Supplementary information

**Additional file 1.** 3-D rotational image of a 26 years old female with meningoencephalitis and septic shock, 170 cm, 59 kg. Slice width 0.625 mm. Aortic volume 77 ml, distance form aortic valve to femoral detector 41 cm, CO 3.3 l/min, MTt 17 s, GEDV 502 ml, GEDVI 293 ml/m^2^.

**Additional file 2.** 3-D sagittal rotational image of a 72 years old female with aneurysmal subarachnoid hemorrhage, 165 cm, 78 kg. Slice width 0.625 mm. Aortic volume 211 ml, distance from aortic valve to femoral detector 62 cm, CO 3.3 l/min, MTT 39 s, GEDV 1263 ml, GEDVI 787 ml/m^2^.

## Data Availability

Individual patient data supporting the conclusions is available in the Zenodo repository [38].
